# Induction of Synthetic Polyploids and Assessment of Genomic Stability in *Lippia alba*

**DOI:** 10.3389/fpls.2020.00292

**Published:** 2020-03-26

**Authors:** Sirlei Aparecida Julião, Christiane do Valle Ribeiro, Juliana Mainenti Leal Lopes, Elyabe Monteiro de Matos, Aryane Campos Reis, Paulo Henrique Pereira Peixoto, Marco Antonio Machado, Ana Luisa Sousa Azevedo, Richard Michael Grazul, José Marcello Salabert de Campos, Lyderson Facio Viccini

**Affiliations:** ^1^Department of Biology, Federal University of Juiz de Fora, Juiz de Fora, Brazil; ^2^Department of Botany, Federal University of Juiz de Fora, Juiz de Fora, Brazil; ^3^Embrapa Dairy Cattle Research Center, Juiz de Fora, Brazil; ^4^Department of Chemistry, Federal University of Juiz de Fora, Juiz de Fora, Brazil

**Keywords:** artificial polyploidy, colchicine, essential oil, FISH, flow cytometry, genomic instability, medicinal plants, SSR and ISSR markers

## Abstract

Polyploidy is widely recognized as a major evolutionary force in plants and has been reported in the genus *Lippia* (Verbenaceae). *Lippia alba*, the most studied species, has been documented as a polyploid complex involving at least four ploidal levels. *L. alba* presents remarkable chemical and genetic variation and represents a model for understanding genome organization. Although the economic and medicinal importance of the species has been widely described, no established polyploid induction protocol has been reported so far. Here, we describe the production of synthetic polyploid plants of *L. alba* using colchicine. The ploidal levels were estimated by flow cytometry and chromosome counting. In addition, FISH and molecular markers approaches were used to confirm the stability of the synthetic polyploids. The major component of the essential oils was estimated by GCMS to compare with the natural individuals. Tetraploids and triploids were produced providing new opportunities for investigating medicinal, pharmacological, and economic applications as well as addressing intrinsic questions involved in the polyploidization process in tropical plants.

## Introduction

Polyploidy (whole-genome multipication) is widely recognized as a major evolutionary force in plants (Stebbins, 1971; Grant, 1981; Otto and Whitton, 2000; Soltis and Soltis, 2009, 2012; Aversano et al., 2012; Wendel, 2015; Wendel et al., 2016). It is estimated that polyploidy events occurred in all angiosperms (Jiao et al., 2011; Albert et al., 2013). Changes in ploidal level may result in broad phenotypic modifications (e.g., Ramsey and Schemske, 2002; Soltis et al., 2003; Adams and Wendel, 2005). These changes may provide polyploids with short-term adaptive potential (Van de Peer et al., 2017) and the opportunity to exploit new niches (e.g., Marchant et al., 2016). In addition, synthetic polyploids have been largely employed to increase agronomic traits mainly due to its higher physiological and morphological fitness compared to their natural diploids (Chung et al., 2017; Cui et al., 2017; Salma et al., 2017; Wei et al., 2018).

Polyploidy events have been documented in the genus *Lippia* (Verbenaceae) (Viccini et al., 2005; Campos et al., 2011; Pierre et al., 2011). *Lippia alba* (Mill.) N. Brown (Linnaeus), the most studied species, has been described as a polyploid complex involving at least four ploidal levels (2*n* = 30, 45, 60, and 90) (Pierre et al., 2011; Reis et al., 2014). An interesting association between ploidal level and chemical profile of essential oil was also reported. Diploid and tetraploid accessions are citral producers while triploid accessions are linalool producers (Viccini et al., 2014). Genetic distance by molecular markers and phylogenetic analysis showed that natural accessions grouped by ploidal level and only one origin of triploids (Lopes et al., 2019, in press).

*Lippia alba* is a tropical species widely distributed throughout the Americas. In Brazil, the species is found all over the country (Reis et al., 2014). *L. alba* has been characterized by its remarkable morphological, phytochemical and genetic variations (Manica-Cattani et al., 2009; Jezler et al., 2013; Reis et al., 2014), as well as its economic importance and medicinal properties (Hennebelle et al., 2008). Biological characteristics such as toxicity repellency, antifungal, antibacterial, and antioxidant properties have been identified in essential oils or extracts of *L. alba* that are widely used in folk medicine (Glamočlija et al., 2011; Chies et al., 2013; Peixoto et al., 2015).

Considering the medicinal importance of *L. alba* and the extraordinary chemical and genetic variation, the species represents a model for understanding genome organization, the origin of a natural polyploid, and its association with chemical profile variation. Thus, the production of synthetic polyploids constitutes a great opportunity for replicating the natural polyploidization process in the *L. alba* complex (Münzbergová, 2017; Pavlíková et al., 2017; Castro et al., 2018), opening a window for understanding polyploidization in the tropics as well as for increasing the production of essential oil of economic interest.

Genome doubling is usually induced by compounds that interfere with cell division and are either applied to *ex vitro* or *in vitro* plants (Dhooghe et al., 2011). Colchicine is the oldest and most widely used compound for polyploidization induction (Nebel, 1937; Niel and Scherrmann, 2006; Dhooghe et al., 2011).

Although many studies provide polyploidization protocols (Rêgo et al., 2011; Gomes et al., 2014; Tavan et al., 2015; Widoretno, 2016; Salma et al., 2017; Denaeghe et al., 2018), they generally demonstrate that the optimal procedures are rather species specific. The development of a proper method for polyploidization requires the conduction of several tests to obtain the most suitable combination of antimitotic agent, concentration, exposure time, type of explants, exposure method, and the regeneration mode employed (Allum et al., 2007; Dhooghe et al., 2011; Yang et al., 2014; Sattler et al., 2016). Therefore, the success of induction depends on the way each procedure is performed in each phase and the correct interaction of each step.

In spite of the economic and medicinal importance of *L. alba* (Hennebelle et al., 2008; Reis et al., 2014; Viccini et al., 2014; Lopes et al., 2019, in press) no established polyploidization protocol for the species has been reported so far. Here, we describe the production of synthetic polyploid plants of *L. alba.* We also tried to broadly understand the consequence of the polyploidization process, addressing the following questions about the new plants obtained: (1) Are the ploidal levels and chromosome numbers the same in all synthetic plants? (2) Do synthetic plants remain stable over time after polyploidy induction? (3) Do synthetic plants have the same chemical profile as the natural ones? We hope our study of these synthetic polyploids provides an opportunity for industry exploration, to discover new biological activities as well as to disclose the evolutionary process immediately following polyploidization, which may be involved in the formation of natural polyploids.

## Materials and Methods

### Plant Material and *in vitro* Propagation

A diploid accession of *L. alba* (2*n* = 2*×* = 30) was used for chromosome duplication. The accession belongs to the *L. alba* collection of the Universidade Federal de Juiz de Fora, Minas Gerais, Brazil (voucher number 48374, deposited at Herbarium Leopoldo Krieger CESJ-UFJF). *In vitro* plantlets were maintained in test tubes containing 15 mL of MS-based medium devoid of growth regulators and subcultivated at intervals of approximately 40 days. The culture was kept at 25 ± 1°C under a light regime of 16/8 h (hours) (light/dark) cycle of 35 μmol m^–2^ s^–1^ illumination provided by cool, white fluorescent tubes.

### Polyploidy Induction and Acclimatization of Plantlets

A pilot experiment was performed to determine the most efficient treatment for polyploidy induction (Supplementary Table S1). As a result, two concentrations of colchicine were chosen (0.2% and 0.02%) during 4 h and 72 h of exposure. A colchicine-free MS medium was used as control.

Both for the pilot and the final experiment, nodal segments of *L. alba* were inoculated in colchicine MS medium. At the end of the exposure time, the explants were washed three times with autoclaved distilled water, and then inoculated in a colchicine-free MS medium. For each treatment, one hundred explants were inoculated in a completely randomized design. The treatments were composed of four replicates containing twenty-five explants each.

All plants that survived were *in vitro* propagated for 40 days. Individuals from 3 to 7 cm high were acclimated. Approximately 30 days later, the plants were transplanted into 10 L vessels containing soil and substrate mixture BioPlant (3:1).

### Determination of Ploidal Levels

We evaluated the ploidal level of regenerated plantlets in two moments. At first, flow cytometry was performed using *in vitro* plantlets after 40 days of culture. The second evaluation was done when the plants reached 18 months, in the greenhouse.

For DNA content estimation, around 25 mg of leaves and roots were chopped in a Petri dish containing 1 mL of cold ice LB01 buffer (Galbraith et al., 1983). The suspension of nuclei was filtered and stained with 25 μl of propidium iodide (10 mg L^–1^) (Sigma). 2.5 μl of RNA seq (20 mg L^–1^) (Sigma) was added to each sample (Dolezel et al., 2007). At least 10,000 nuclei were analyzed per sample using a FACSCanto II flow cytometer (Becton Dickinson). FACS software Diva 6.1.3 was used to build the histograms that were analyzed using Flowing software 2.5.1 (available at http://www.flowingsoftware.com/). The ploidal level was checked taking as reference the position of the G1 peak of the diploid plant. Three estimates were made for each plant.

The chromosome counting, 45S rDNA mapping, molecular and chemical analyses were performed when the plants were established in the greenhouse.

### Chromosome Counting and 45S rDNA Mapping

In order to verify the chromosome number and confirm the ploidal level of synthetic plantlets, root tips were pretreated with 0.003M of 8-hydroxyquinoline (Sigma) at 4°C for 9 h and fixed in ethanol: acetic acid (3:1). The radicular meristems were submitted to enzymatic maceration in 20% pectinase (Sigma) and 2% cellulase (Serva-Onozuka R-10) at 37°C for 6 h. Cytological preparations were performed according to Carvalho and Saraiva (1993).

The fluorescence *in situ* hybridization (FISH) was performed as described by Jiang et al. (1995). The hybridization mixture was denatured at 85°C for 10 min and immediately transferred to an icebox. The slides were denatured at 85°C for 1 min and treated with a series of alcohol washes (70, 90, and 100% ethanol for 5 min each). The hybridization mixture was then added to the slides and the chromosomes allowed to hybridize at 37°C for 18 h in a humidified chamber. Post-hybridization washes were carried out using 2 × SSC buffer (0.3 mol L^–1^ sodium citrate, 0.03 mol L^–1^ sodium chloride, pH 7) and 1 × PBS buffer (0.136 mol L^–1^ sodium chloride, 0.27 mol L^–1^ potassium chloride, 0.1 mol L^–1^ dibasic sodium phosphate, 0.2 mol L^–1^ monobasic potassium phosphate, pH 7.4). Probes were detected with anti-DIG conjugate with rhodamine (Sigma) and post-detection washes were performed using 1 × TNT buffer (0.1 mol L^–1^ Tris, 0.15 mol L^–1^ sodium chloride, 0.05% Tween-20) and 1 × PBS at room temperature. Chromosomes were counterstained with 2 μg mL^–1^ of DAPI (Sigma). The slides were mounted in Vectashield (Vector, Burlingame, CA, United States). Signal visualization was performed under Olympus BX53 fluorescence microscopy and the images were photographed with an Olympus DP72 camera attached to the microscope.

### DNA Extraction, Amplification, and Data Analysis

Total genomic DNA from the leaves plants established in the greenhouse was extracted using the CTAB method (Doyle and Doyle, 1990) with a minor modification (double extraction with chloroform-isoamyl alcohol). After extraction, the DNA of the samples were solubilized and quantified using a Nanodrop Spectrophotometer (ThermoFisher Scientific, Inc., Wilmington, DE, United States), diluted to 25 ng μl^–1^, and kept at −20°C for subsequent use.

Eleven ISSR primers (Supplementary Table S2) and seven SSR primers (Supplementary Table S3) were selected to estimate the genetic profile of diploid and synthetic polyploid plants. The SSR primers were synthesized with M13-tailed forward primers (Supplementary Table S3). PCR was carried out in a DNA Thermal Cycler Mastercycler^®^ (Eppendorf-Netheler-Hinz GmbH, Hamburg, Germany) following the specifications given in Supplementary Tables S2, S3.

For ISSR analysis the amplification fragments were analyzed on 2% agarose gels. The products of PCR amplification were recorded as a binary matrix, in which the presence or absence of similarly sized fragments were marked 1 or 0, respectively. Only consistent and reproducible bands between 200 and 800 bp of size were included in the analysis.

The analysis of allelic polymorphisms was performed comparing polymorphic bands between the mother plant and synthetic polyploids. SSR fluorescent products were detected by capillary electrophoresis using a MegaBACE1000 sequencer (GE Healthcare, Buckinghamshire, United Kingdom). The SSR fragment size was measured using the Fragment Profile program (GE Healthcare, Buckinghamshire, United Kingdom). All individuals were scored for the presence or absence of SSR alleles at each of the seven loci. The data were entered into a binary matrix as discrete variables, 1 for presence and 0 for absence of the allele, and this data matrix was subjected to genetic distance analysis.

The Jaccard and Dice coefficients were used to generate dendrograms for ISSR and SSR data using the Unweighted Pair Group Method with Arithmetic Means (UPGMA) and to estimate the similarity values among accessions. The genetic distance and bootstrap analyses of the data were performed with 1000 repetitions using Ntsys v2.11 software (Rohlf, 2000) and PAST (Hammer et al., 2001). As the ISSR and SSR dendrograms showed the same result, they were performed together.

The genetic profile was investigated using Bayesian inference clustering as implemented in Structure 2.3.4^1^ (Pritchard et al., 2000), and final plots were produced in Structure Plots v2.0 (Ramasamy et al., 2014). We analyzed 12 accessions as dominant data, coded as presence/absence, using the admixture model with uncorrelated allele frequencies (Stift et al., 2019). The Monte Carlo Markov Chain was run for 100,000 steps, following a burn-in of 10,000 steps. Simulations were performed for the number of groups (*K*) varying from 1 to 10. We used Structure Harvester (Earl and VonHoldt, 2012) to calculate Δ*Km* (Evanno et al., 2005).

### Essential Oil Microextraction

Leaf tissue (approximately 300 mg) from each individual was frozen in glass vials at −18°C for 24 h. After freezing, 0.5 mL of hexane and 0.25 mL of methanol were added to each sample. The samples were kept in a 70 KHz ultrasonic bath (Thornton-INPEC) at room temperature for 1 h to accelerate the extraction process. Subsequently, the supernatant was filtered through a sterile cotton swab. From the clear solution of extracted oils 1 μL was analyzed on a mass spectrometer gas chromatograph (GCMS-QP2010 Plus; Shimadzu). A 30 m × 0.25 mm Rtx-5MS^®^ (Restek) column was used. The oven temperature programming started at an initial temperature of 70°C, maintained for 3 min, followed by an increase of 6°C min^–1^ to 300°C. The injector was operated in split mode (1:10), at 240°C, an interface and the mass detector operated at 300°C. Helium was used as a carrier gas, with a flow of 1.53 mL min^–1^. A standard mixture of linear hydrocarbons was injected under the same conditions of use. Component identification was performed by comparing mass spectra with data from the NIST 9.0 database (correlation > 97%) and confirmed by retention index (Kováts Index) being calculated for each component and compared to data in the literature (Adams, 2007).

## Results

### Polyploidy Induction and Ploidal Stability

**FIGURE 1:**
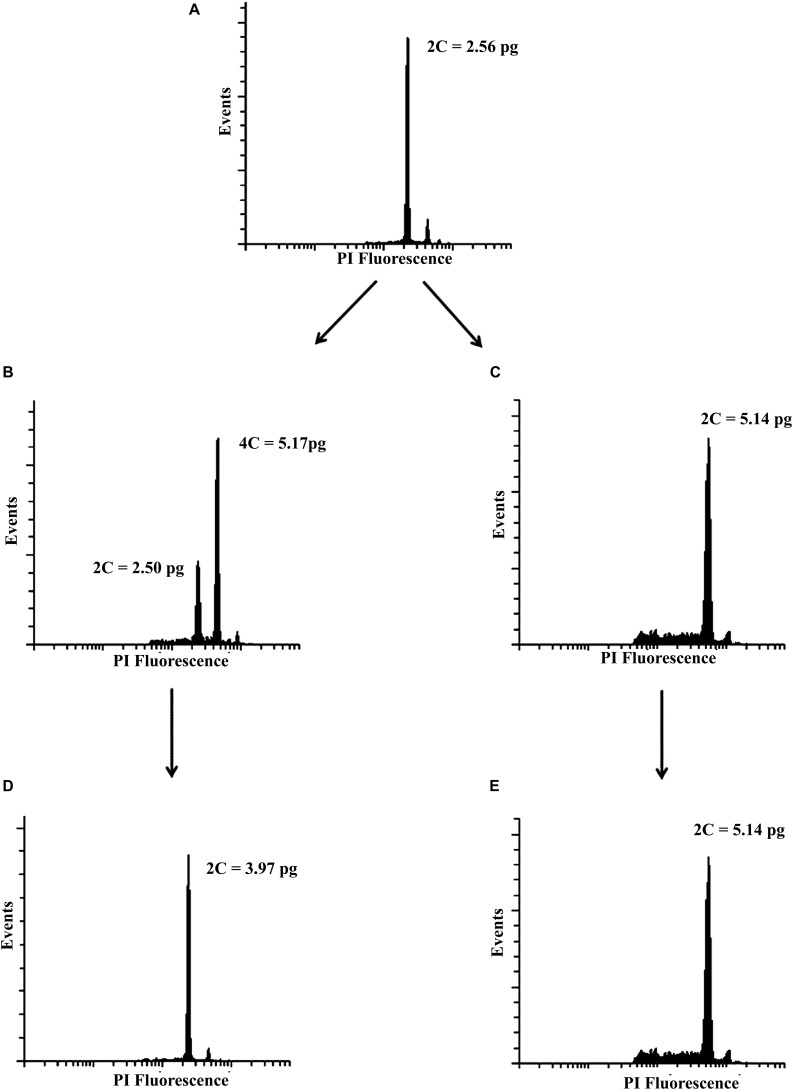
Representative histograms of the mother plant and synthetic polyploids. **(A)** mother plant (diploid), **(B)** mixoploid, **(C)** tetraploid 40 days after polyploidy induction, **(D)** triploid, and **(E)** tetraploid acclimatized in the greenhouse.

Based on the pilot experiment (Supplementary Table S1), we decided to evaluate two concentrations of colchicine, 0.2% and 0.02% during 4 h and 72 h of exposure. Forty days after culture, the ploidal level of regenerated plantlets were assessed. Mixoploids and tetraploids were identified (Figures 1B,C). The treatment of 0.2% colchicine showed the highest effect on the modification of the ploidal level of surviving plants. Five mixoploids were obtained when 0.2% colchicine was applied for 4 h, and one tetraploid was observed when 0.2% colchicine for 72 h was used. Using 0.02% colchicine during 4 h, three mixoploid plants were regenerated (Table 1). For ploidy determination, the value of the DNA content of the diploid plant (2.56 ± 0.06 pg) was used as a reference (Figure 1A). The tetraploids showed the double of DNA content observed for diploids (2C = 5.14 ± 0.03 pg). The mixoploids showed two peaks, corresponding to diploid and tetraploid cells (Figure 1B). Synthetic mixoploids and tetraploids were *in vitro* cultivated for 3 months.

**TABLE 1:** Survival rate and ploidal level of *Lippia alba* treated with colchicine at different concentrations and exposure times.

		Explants exposed	Surviving	Tetraploid	Mixoploid
4 h	Control	100	78	–	–
	0.02%	100	58	–	3
	0.20%	100	38	–	5
72 h	Control	100	64	–	–
	0.02%	100	44	–	–
	0.20%	100	11	1	–

After that, the plants were transferred to the greenhouse and their ploidal levels were reassessed. The two peaks previously identified for mixoploids were replaced and only one peak was revealed that showed the same C-value of natural triploids (2C = 3.97 ± 0.03 pg) (Figure 1D). In other words, the mixoploids became triploids. The tetraploids maintained the ploidal level initially estimated (Figure 1E). We assessed a total of five synthetic tetraploids and six synthetic triploids. The flow cytometry analysis confirmed the stability of the ploidal level in two tissues (leaf and root) over 5 years. No chimeras were found during this period (Supplementary Figure S1).

**FIGURE 2:**
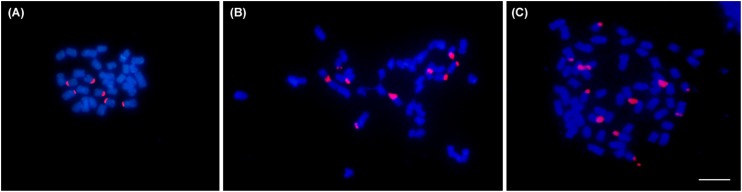
Representative metaphases of three *Lippia alba* cytptypes: **(A)** diploid (2*n* = 30), **(B)** triploid (2*n* = 45), and **(C)** tetraploid (2*n* = 60) individuals. Chromosomes were counterstained with DAPI (blue), 45S rDNA marker was stained with rhodamine (red). Bar = 5μm.

Chromosome counts confirmed 2*n* = 30 for the diploid (mother plant), 2*n* = 60 for tetraploids and 2*n* = 45 for triploids (Figure 2). In addition, some aneuploid cells also were observed in emergent triploids. Their chromosome numbers ranged from 2*n* = 30 to 2*n* = 60. FISH mapping of ribosomal genes revealed six terminal sites for diploid, nine for triploids and 12 for tetraploids (Figure 2).

### Molecular Profile

Fifty-five ISSR loci with an average of five loci per primer were observed. The number of loci ranged from three (UBC-859) to seven (UBC-826 and UBC-857). The fragment size ranged from 200 to 800 bp (Supplementary Table S2). The analysis of allelic polymorphisms was performed comparing the synthetic polyploids (triploids and tetraploids) with the mother plant. The number of polymorphic bands between the mother plant and synthetic triploids ranged from 24 to 31, while between the mother plant and synthetic tetraploids it varied from one to three. Four out of six synthetic triploid plants showed polymorphisms for all primers. The tetraploid plants showed polymorphism for only one or two primers. A high percentage of polymorphism (43.63 to 56.36%) was detected between the mother plant and synthetic triploids. The comparison between the mother plant and the synthetic tetraploids showed lower polymorphism rate, ranging from 1.81 to 5.45%. These polymorphisms are mainly due to the presence of fragments in synthetic polyploid plants that were not observed in the mother plant.

The analysis of SSR loci revealed 25 alleles, with an average of 3.6 alleles per primer. The size of the alleles ranged from 101 to 193 base pairs. The analysis of the allelic polymorphisms was performed as for ISSR markers. The majority of new alleles were observed in synthetic triploids. On average, up to 77% of alleles detected in triploid plants correspond to new alleles, while tetraploid plants revealed 18 to 36% of new alleles.

**FIGURE 3:**
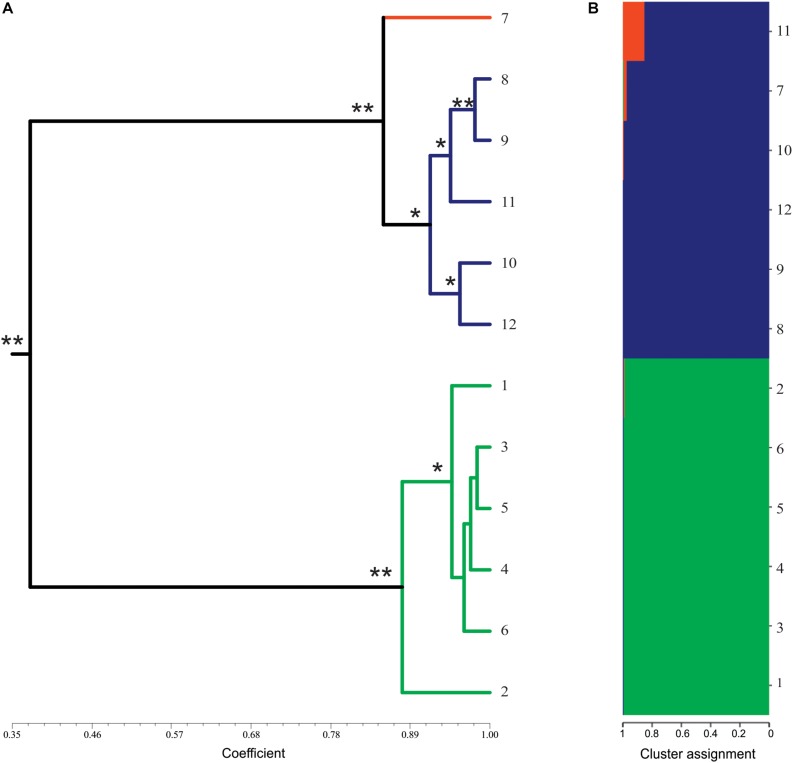
Molecular profile of synthetics plants of *Lippia alba*. **(A)** Dendrogram of genetic similarity by UPGMA of presence/absence of alleles using combined data (ISSR and SSR makers) from 12 plants of *L. alba*. The colors of the branches represent different ploidal levels: diploid in orange, triploid in green, tetraploid in blue. * represents bootstrap values above 50% and ** represents bootstrap values above 90%. Dendrograms with JACCARD and DICE coefficients were identical. **(B)** Bayesian analysis of the genetic structure of 12 plants of *L. alba*. The colors represent the proportion of the genome shared for each individual. Similar genomes are represented by the same color.

The analysis of genetic similarity was done separately for ISSR and SSR (data not shown) and showed similar results. Therefore, the analysis was performed together. The similarity coefficients ranged from 0.35 to 0.98 (average of 0.68). Based on the similarity index, two clusters were formed: one with the mother plant and synthetic tetraploids, and another with only synthetic triploid plants. The similarity index among the mother diploid plant and the synthetic tetraploids varied from 0.84 to 0.98 (average of 0.93). Regarding the triploids, the coefficient of similarity among them ranged from 0.85 to 0.98 (average of 0.95). Considering all plants analyzed, the lowest similarity value (0.346) was observed between tetraploid and triploid plants, and the highest (0.982) was observed among triploid plants (Figure 3A).

The Structure analysis using Δ*Km* (Evanno et al., 2005) method indicated that the best number of groups is *K* = 3. This analysis revealed the genetic structure among the natural diploid and synthetic plants (Figure 3B). The triploid plants seem to be distinct from the other ploidal levels while the tetraploids showed a genomic structure similar to the mother plant (Figure 3B). These results are similar to those observed using the similarity analysis (Figure 3A).

### Essential Oil Profile

**FIGURE 4:**
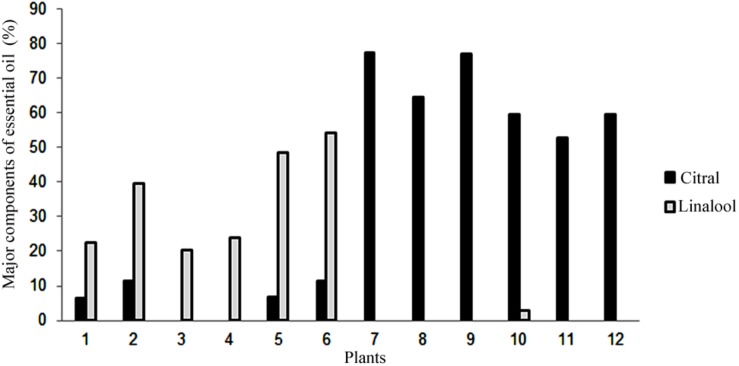
Percentage of the major essential oil content detected by gas chromatography. Mother diploid plant (7), synthetic triploid plants (1–6) and synthetic tetraploid plants (8–12). Gray bars, Citral; Black bars, Linalool.

Using gas chromatography coupled to a mass spectrometer, it was possible to identify the components of the essential oil of synthetic polyploid plants of *L. alba*. The main constituents detected were citral (neral and geranial) and linalool (Figure 4). The citral was the major component essential oil of the natural diploid (77.51%) and synthetic tetraploids (from 52.71 to 77.04%). The linalool constituent was detected as a major component in all synthetic triploid plants, ranging from 20.3 to 54.13% (Figure 4).

## Discussion

The induction of polyploids has been widely used as a strategy to investigate the effects of artificial genomic duplication on several plant species. Morphological, histological, physiological, agronomic, and genomic traits have been evaluated in different studies (Adams and Wendel, 2005; Buggs et al., 2008; Rêgo et al., 2011; Hegarty et al., 2013; Gomes et al., 2014; Tavan et al., 2015; Gao et al., 2016; Iannicelli et al., 2016; Yan et al., 2016; Sadat-Noori et al., 2017; Salma et al., 2017; Zhou et al., 2017).

Although polyploidy induction has been widely recognized as an important strategy for chromosome duplication in plants, the protocols are still associated with low efficiency. Here we reported the first attempt to produce synthetic polyploids in *L. alba*. The pilot experiment sought to screen interactions between colchicine concentrations and exposure times. We obtained the higher survival rates when lower colchicine concentrations and shorter exposure times were employed. The same effect was previously reported for other species, and was mainly attributed to the toxic effect of colchicine (Niel and Scherrmann, 2006; Lehrer et al., 2008; Sajjad et al., 2013).

The number of polyploid plants here obtained suggests that the 0.2% colchicine concentration combined with 4 h exposure time was the most successful treatment. This opens good perspectives for new trials on testing the procedure using plants at different stages of development as well different ploidy, in particular if we consider that *L. alba* has naturally at least four ploidal levels which have been described (Reis et al., 2014).

The ploidal level of synthetic polyploid plants should be periodically checked to ensure the maintenance of the ploidal level (Väinölä, 2000; Harbard et al., 2012; Blasco et al., 2015). The analyses of flow cytometry, chromosome counting, genetic and chemical diversity are important for identifying possible phenotypic and genotypic variations in synthetic polyploids. Here we observed that tetraploids kept their ploidal level, while mixoploids did not. Stable synthetic tetraploids have been reported in *Eriobotrya japonica* (Thunb.) Lindl (Blasco et al., 2015) and in *Rhododendron* L (Väinölä, 2000). On the other hand, instability of synthetic tetraploid plants was observed in *Acacia mangium* Willd. Two tetraploid plants were reclassified as diploids and two as mixoploid 16 months later, when the plants were transferred from the greenhouse to the field (Harbard et al., 2012).

These results reveal that the response to the duplication process may vary among species, according to the methodology employed and the maintenance of the synthetic plants. Vanstechelman et al. (2010) suggest that synthetic polyploid plants should be reanalyzed after the *in vitro* micropropagation procedure. According to the authors, many sectoral chimeras are not detected in the first analysis by flow cytometry. Plants of *Spathiphyllum wallisii* Regel, for instance, that were initially classified as tetraploids showed roots with diploid and/or mixoploid cells (Vanstechelman et al., 2010). Here, the synthetic tetraploids of *L. alba* presented sixty chromosomes in the metaphases confirming the ploidal level indicated by the flow cytometry analysis. Chromosome mapping of tetraploids showed that the number of 45S rDNA increases proportionally, revealing that the protocol was able to duplicate the genome with no evidence of chromosomal rearrangements after the duplication. The synthetic tetraploids grouped with the mother diploid plant with high genetic similarity. Similar results were observed when a larger number of natural diploid and tetraploid accessions were studied together using microsatellites and phylogenetic inferences (Lopes et al., 2019, in press). On the other hand, FISH mapping of ribosomal genes in natural tetraploids of *L. alba* previously revealed only eight 45S sites (Reis et al., 2014). The difference between natural and synthetic tetraploids regarding the number of 45S markers can be attributed to the structural alterations and genome downsizing, frequently reported in natural polyploids (Hegarty et al., 2013; Doyle and Coate, 2019).

Although synthetic tetraploids showed a similar karyotype compared to the natural diploid, the mixoploid individuals revealed a different scenario. Interestingly, when the DNA content of mixoploids was reassessed, we realized that the plants presented an intermediary DNA amount between diploid and tetraploid, that was equivalent to the natural triploids. Besides, the metaphases of synthetic triploids had 2*n* = 45 chromosomes, as observed for natural triploids of *L. alba*, suggesting that some chromosomes were lost in tetraploid cells. Losses of whole chromosomes can occur in regenerated mixoploids in attempt to stabilize the genomic constitution after polyploidy induction in the short-term, but the emergence of a new ploidal level seems to be rare (Dodsworth et al., 2016; Regalado et al., 2017; Salma et al., 2017; Doyle and Coate, 2019). In addition we could not cannot discard the possibility that some cells 2*n* = 45 could be present in small quantity in mixoploids and this ploidal level increased in detriment of the others after the acclimatization in greenhouse. More studies need to be done for a better understanding of how the emergence of the synthetic triploids occurs.

Commonly, the polyploid induction may result in chimeras, that typically became stable at one ploidal level. However, some individuals maintain the mixoploidy state (Harbard et al., 2012; Eng and Ho, 2019). In *L. alba*, one natural mixoploid individual was described previously, but chromosome losses were not notified in this accession and the mixoploidy was stable over time (Pierre et al., 2011). Curiously, this mixoploid had cells with chromosome number ranging from 2*n* = 12 to 60, being 44, 45, and 46 the most frequent numbers of chromosomes observed (Pierre et al., 2011).

Here we observed the majority of aneuploid cells in synthetic triploids varing from 2*n* = 38 to 47, but they were restricted to a few metaphases. These cells may be remnants of the previous mixoploidy state. This fact might be linked to a putative process of karyotype uniformity and these cells possibly have some advantage over diploid cells. Although it can be considered a rare event, the emergence of triploid plants using a protocol to produce synthetic tetraploids has been previously described. In *Pyrus communis* L., triploid plants were obtained after *in vitro* treatment of leaf explants with colchicine (Sun et al., 2009). The treatment of apical meristems with colchicine generated triploid plants of poplar (Ewald et al., 2009). The germinated seedlings of trifluralin-treated *Rosa chinensis* Jacq. yielded triploid (2*n* = 3*×*) and aneuploid (2*n* = 3*×*−1) plants (Zlesak et al., 2005). None of the authors above explained the emergence of triploid plants obtained during tetraploid induction treatments.

In addition to the numerical variations, structural rearrangements are frequently reported in recently formed polyploids and such alterations can originate new allelic polymorphisms among individuals (Tayalé and Parisod, 2013). The triploids showed the highest polymorphism rates and new alleles when compared to the mother diploid. The DNA elimination and a genomic shocking (Buggs et al., 2008) might explain these results. The genomic reorganization detected by molecular markers has also been identified in *Chrysanthemum lavandulifolium* (Fisch. ex Trautv.) Makino, *Paspalum notatum* Flüggé, *Citrullus lanatus* (Thunb.) Mansf., and *Eragrostis curvula* (Schrad.) Nees (Martelotto et al., 2007; Mecchia et al., 2007; Wang et al., 2009). On the other hand, the new synthetic tetraploids showed similar molecular profile when compared to the mother plant (diploid), corroborating the cytogenetic data. Similar results have been observed in *Solanum commersonii* Dunal, *Solanum bulbocastanum* Dunal, and in *Citrus limonia* (L.) Osbeck (Allario et al., 2011; Aversano et al., 2013, 2015). The distinctiveness of the triploids and the similarity of the tetraploids comparing with the diploid was also detected by genetic similarity analysis. Interestingly, the same scenario was previously observed for natural triploids when the molecular profile, the essential oils production, and the morphology were assessed. Triploids seem to be particularly different comparing with other ploidal levels (Viccini et al., 2014; Lopes et al., 2019 in press).

Regarding the essential oil production, the synthetic tetraploids produced the same major component of the essential oil that was produced by the mother plant, while the triploids changed the major component to linalool. The synthetic polyploids showed the same profile observed for the natural polyploids (Viccini et al., 2014). The analysis of the metabolism of autopolyploid plants suggests that polyploidy may cause both qualitative and quantitative changes in the essential oil, due to changes in the mechanisms that regulate its biosynthesis (Fasano et al., 2016; Iannicelli et al., 2020). Vieira et al. (2016) suggested that synthesis of citral, geraniol and other compounds prevailed in diploids, while non-oxygenated monoterpenes were the major ones in polyploids of *Citrus limonia*. Hannweg et al. (2016) also identified changes in major chemical components of essential oils due to the polyploidy induction in *Tetradenia riparia* (Hochst.) Codd (Lamiaceae), a close related family of Verbenaceae. The metabolic activity may be increased due to alteration of gene expression or changes in the concentration of the secondary metabolites (Fasano et al., 2016; Iannicelli et al., 2020). Evidence of the genetic duplication affecting metabolic profiles of different plant species has been widely reported (Caruso et al., 2011; Dehghan et al., 2012; Trojak-Goluch and Skomra, 2013; Gomes et al., 2014; Xu et al., 2014; Tavan et al., 2015; Iannicelli et al., 2016) which reinforces the application of genome duplication protocols for manipulating the biosynthesis of compounds of economic interest.

## Conclusion

The polyploidization protocol was able to produce stable polyploids in *L.alba*. The synthetic tetraploid showed the same ploidal level over time with similar molecular, karyotipic, and chemical profiles. Interestingly, the mixoploids underwent karyotype uniformity, and the majority of their cells showed 45 chromosomes. Besides that, the emergence of alleles and changes on the major component of essential oil were observed.

The production of synthetic polyploids enables comparison with the natural polyploidization process. Considering all loci are potentially homozygous it is possible to infer how the increase in genome size affects the phenotype.

Taking into account that the species is an aromatic shrub with pharmacological and economic applications, those synthetic plants may open a new scenario for manipulating the genome, regarding the gene expression profile or the production of secondary metabolites of commercial interest (Castro et al., 2018; Long et al., 2019).

Few examples of polyploid complexes from the tropics have been documented (Rice et al., 2019), which makes *L. alba* a potential species for studying the polyploidization process in non-model plants.

## Data Availability Statement

All datasets generated for this study are available on request to the corresponding author without restriction.

## Author Contributions

SJ, CR, JC, and LV conceived and designed the experiments. SJ, CR, and PP performed the *in vitro* tissue culture and chromosome doubling experiments. EM and JC carried out the flow cytometry analyses. AR performed the cytogenetic analyses. JL, MM, and AA performed the molecular markers analyses. RG analyzed the essential oil profile. SJ, EM, JL, AR, and LV contributed to the writing of the manuscript. LV revised the manuscript. All authors listed have made a substantial, direct and intellectual contribution to the work, and approved the manuscript.

## Conflict of Interest

The authors declare that the research was conducted in the absence of any commercial or financial relationships that could be construed as a potential conflict of interest.

## Footnotes

1 http://pritchardlab.stanford.edu/structure.html
