# Supplementary material for: Induction of Synthetic Polyploids and Assessment of Genomic Stability in Lippia alba
Source: Front Plant Sci. 2020 Mar 26;11:292. doi: 10.3389/fpls.2020.00292 (PMC7113378; doi:10.3389/fpls.2020.00292)
Supplement: Supplementary file 1 [file Table_1.doc]

Table S1. Pilot experiment, survival rate and ploidal level of *Lippia alba* treated with colchicine at different concentrations and exposure times

| **Treatment** | **Survival (%)*** | **Number of tetraploid plants** | **Number of mixoploid plants** |
| --- | --- | --- | --- |
| Control 2h | 90 | - | - |
| 0.5% 2h | 15 | 1 | - |
| Control 4h | 100 |  |  |
| 0.5% 4h | 35 | - | 1 |
| 0.2% 4h | 50 | 3 | 2 |
| Control 16h | 80 | - | - |
| 0.2% 16h | 66 | - | - |
| 0.1% 16h | 70 | - | 1 |
| Control 72h | 85 | - | - |
| 0.05% 72h | 100 | - | - |
| 0.003125% 72h | 90 | - | 1 |
| Control 34 days | 90 | - | - |
| 0.003125% 34 days | 80 | - | 1 |
| 0.001563% 34 days | 60 | - | 1 |

*Survival was calculated 20 days after inoculation into colchicine-free medium.
