# Supplementary material for: Induction of Synthetic Polyploids and Assessment of Genomic Stability in Lippia alba
Source: Front Plant Sci. 2020 Mar 26;11:292. doi: 10.3389/fpls.2020.00292 (PMC7113378; doi:10.3389/fpls.2020.00292)
Supplement: Supplementary file 2 [file Table_2.doc]

Table S2. Characteristics of eleven ISSR primers in synthetic plants of *Lippia alba* and PCR details.

| Loci | Sequences | *Ta* °C | Size (bp) |
| --- | --- | --- | --- |
| UBC-818 | CACACACACACACACAG | 51 | 300-600 |
| UBC-826 | ACACACACACACACACC | 53 | 200-700 |
| UBC-827 | ACACACACACACACACG | 53 | 300-600 |
| UBC-829 | TGTGTGTGTGTGTGTGC | 55 | 400-800 |
| UBC-830 | TGTGTGTGTGTGTGTGG | 53 | 200-500 |
| UBC-835 | AGAGAGAGAGAGAGAGYC | 50 | 200-500 |
| UBC-841 | GAGAGAGAGAGAGAGAYC | 49 | 300-800 |
| UBC-847 | CACACACACACACACARC | 53 | 400-800 |
| UBC-854 | TCTCTCTCTCTCTCTCRG | 50 | 300-700 |
| UBC-859 | TGTGTGTGTGTGTGTGRC | 55 | 300-500 |

*Ta* annealing temperature; *^a^* primers were designed using Primer 3 (<http://bioinfo.ut.ee/primer3-0.4.0/>) based on the Genbank sequence, the sequences were published before the paper Santos et al., 2012.

PCRs were carried out in 25 µL volume: 1X GoTaq® buffer, 0.5 mM primer, 1.5 mM MgCl2, 0.15 mM dNTPs, 1 unit of Taq DNA polymerase and 30 ng of genomic DNA of *Lippia alba* accessions. The amplification cycles were initial denaturation 94 °C for 4 min and 45 cycles of 94 °C for 1 min, annealing temperature for 45 s, 72 °C for 2 min and the final extension at 72 °C for 7 min.
