# Supplementary material for: Induction of Synthetic Polyploids and Assessment of Genomic Stability in Lippia alba
Source: Front Plant Sci. 2020 Mar 26;11:292. doi: 10.3389/fpls.2020.00292 (PMC7113378; doi:10.3389/fpls.2020.00292)
Supplement: Supplementary file 3 [file Table_3.doc]

Table S3. Characteristics of seven microsatellite primers in synthetic plants of *Lippia alba* and PCR details.

| Loci | Size (bp) | *Ta °*C | Reference |
| --- | --- | --- | --- |
| p60 | 153-167 | 57 | Lopes et al., unpublished |
| p077 | 182-193 | 56 | Lopes et al., unpublished |
| p229 | 112-186 | 50 | Lopes et al., unpublished |
| P440 | 112-191 | 50 | Lopes et al., unpublished |
| p432 | 101-180 | 56 | Lopes et al., unpublished |
| p473 | 105-170 | 59 | Lopes et al., unpublished |
| p484 | 172-176 | 55 | Lopes et al., unpublished |

M13 tag sequence (5’-GTAAAACGACGGCCAGT-3’); *Ta* annealing temperature; *^a^* primers were designed using Primer 3 (<http://bioinfo.ut.ee/primer3-0.4.0/>) based on the Genbank sequence, the sequences were published before the paper Santos et al., 2012.

PCRs were carried out in 10 µL volume: 1X GoTaq® buffer, 0.013 mM forward, 0.5 mM reverse and universal FAM or HEX-labeled M13 primers, 1.5 mM MgCl_2_, 0.2 mM dNTPs, 1 unit of Taq DNA polymerase and 30 ng of genomic DNA of *Lippia alba* accessions. The amplification cycles were initial denaturation 94 °C for 3 min and 35 cycles of 94 °C for 45 s, annealing temperature for 45 s with touch down, 72 °C for 45 s and the final extension at 72 °C for 20 min.
