# Supplementary material for: Induction of Synthetic Polyploids and Assessment of Genomic Stability in Lippia alba
Source: Front Plant Sci. 2020 Mar 26;11:292. doi: 10.3389/fpls.2020.00292 (PMC7113378; doi:10.3389/fpls.2020.00292)
Supplement: Supplementary file 4 [file Data_Sheet_1.PDF]

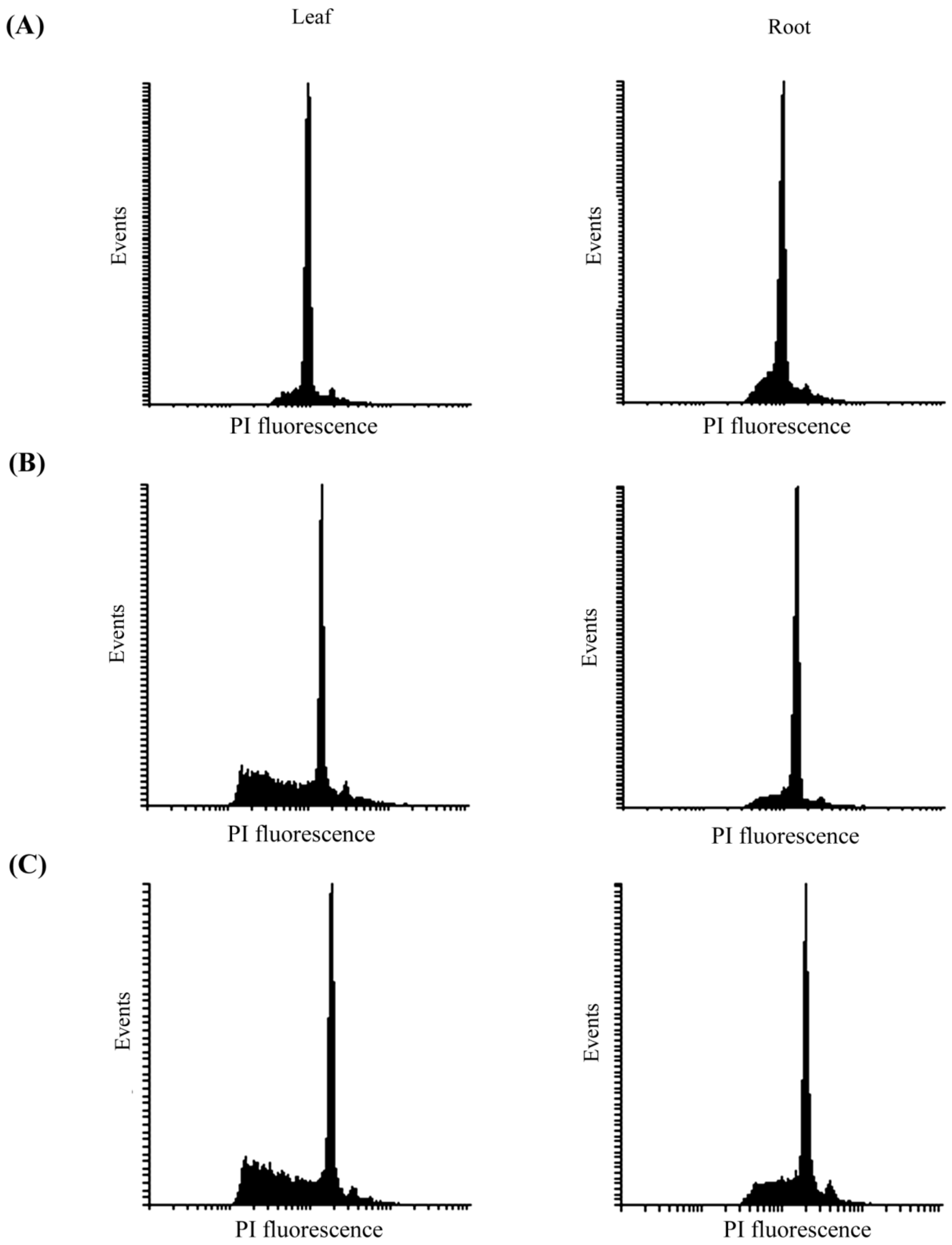

Figure S1. Representative histograms of leaf and root nuclei of the mother plant and synthetic polyploids. The x-axis represents the intensity of fluorescence emitted by the nuclei and the y axis the number of nuclei analyzed. (A) mother plant (diploid), (B) synthetic triploid , (C) synthetic tetraploid.
